# Effect of lyophilized chive (*Allium wakegi* Araki) supplementation to the frying batter mixture on quality attributes of fried chicken breast and tenderloin

**DOI:** 10.1016/j.fochx.2022.100216

**Published:** 2022-01-17

**Authors:** Sin-Young Park, Hack-Youn Kim

**Affiliations:** Department of Animal Resources Science, Kongju National University, Chungnam 32439, Republic of Korea

**Keywords:** Chicken meat, Deep-frying, Lyophilized chives, Physicochemical properties, Sensory characteristics

## Abstract

Batter mixtures for frying chicken breasts and tenderloins were supplemented with different amounts (0, 3, 5, and 7%) of lyophilized chives (*Allium wakegi* Araki). The viscosity of the batter mixture, crispness of the fried batter, fat and ash contents, caloric value, coating pickup, and frying yield were directly proportional, whereas the lightness, redness, yellowness, and pH were inversely proportional, to the amount of lyophilized chives in the batter. Principal component analysis revealed that the aromatic profiles varied between the 0%, 3%, and 5% lyophilized chive-supplemented groups in both the breast and tenderloin samples. However, the aromatic profiles of the 7% and 5% lyophilized chive-supplemented samples were similar. The taste profile of the 7% lyophilized chive-supplemented sample was different from those of the 0%, 3%, or 5% lyophilized chive-supplemented samples. The sensory characteristics of the 5% lyophilized chive-supplemented breast samples and 3% or 5% lyophilized chive-supplemented tenderloin samples received the best scores by sensory panelists.

## Highlights

• As a condiment vegetable ingredient, lyophilized chive was used for the frying batter.
• Improved the physical properties (viscosity and crispness) of the batter mixture.
• Improved coating pickup and frying yield, which are related to economy in deep-frying products.
• Positively affected to flavor and taste of fried chicken breast and tenderloin.

## Graphical abstract

**Figure:**
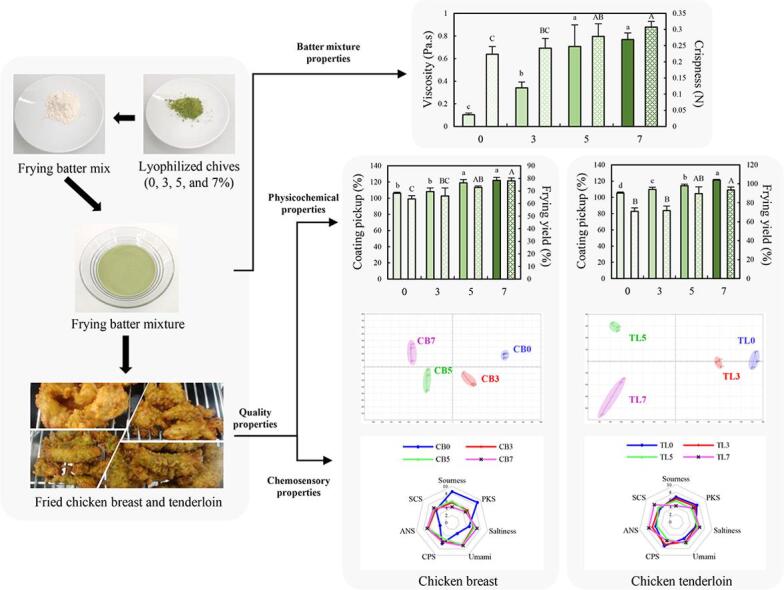

## 1 Introduction

Deep-frying is a very common cooking method used across the globe and deep-fried foods are widely consumed because of their characteristic flavor. Oil extracted from soybean, corn seed, canola, olive, etc. are used in this cooking process. The method of deep-frying varies according to the type of food being fried. Generally, the raw material is coated with batter and fried in edible fat, which is maintained at a high temperature, for a fixed duration (Adedeji & Ngadi, 2011). During this process, moisture evaporates from the batter creating spaces where oil can be absorbed. This fat absorption by the food imparts a unique flavor and taste to fried foods and consequently enhances their sensory characteristics (Sahasrabudhe, Rodriguez-Martinez, O'Meara, & Farkas, 2017). Additionally, the batter, which is exposed to the high temperature, rapidly hardens. This prevents moisture exudation from the raw material and subsequently generates a crispy outer texture. Thus, the batter is an important factor in determining the quality of fried foods (Varela & Fiszman, 2011).

The batter used for frying is distributed in the market as a batter mix that consists of various ingredients. The batter mix contains small amounts of additives (salt, sugar, black pepper, etc.), including spices to enhance the taste and flavor, but it mostly consists of wheat, corn, and rice flour. The main characteristic of the grain flour added to the batter mix is its high rehydration rate. When mixing the batter mix with water, the flour is hydrated and forms a viscous colloidal mixture (Yang, Li, Li, Sun, & Guo, 2020). Therefore, the composition and ratio of ingredients of the batter mix are important determinants of its quality. Recently, the diversification of consumer consumption patterns has led to the development of batter mixes, with various condiment vegetables, that give deep-fried foods a variety of flavors and tastes. For example, batter mixes with pepper or chili powder impart a spicy taste, whilst those with curry powder impart a unique flavor (Carvalho et al., 2018, Rao et al., 2017). However, despite the existence of various condiment vegetables, only a few varieties of already widely popular spices are used in cooking. Therefore, with the increase in consumer demand for different tastes and aromas in the food industry, it has become necessary to use new spices in foods.

Chives (*Allium wakegi* Araki) is a hybrid of spring onions (*Allium fistulosum* L.) and onions (*Allium ascalonicum* L.). Chives exhibit the antioxidant, anti-cancer, and antibacterial properties that are functionally characteristic to the genus *Allium* (Bhandari et al., 2017, Suleria et al., 2015). Furthermore, chives promote lipid metabolism and are rich in fiber, which aids digestion and absorption within the body (Lee et al., 2009). The phenotype and functional properties of chives are similar to those of spring onions. However, chives have a relatively spicier taste and stronger flavor compared to spring onions. Due to these characteristics, chives can impart a characteristic spicy taste and strong flavor to food. Chives can be used as a seasoning or garnish, and can be added to sauces (Kook, Kim, & Chung, 2020). Therefore, these characteristics of chives are proposed to give a positive flavor and taste when they used in meat products.

Condiment vegetables are optimally used in a powdered form during the industrial preparation of various foods. Among the methods of powdering of plant materials, lyophilization (freeze-drying) can minimize the loss of nutrients from plant materials. Unlike hot-air drying, which circulates hot air to dry the subject material, lyophilization sublimates moisture under vacuum conditions in a cryogenic state, below –60 °C (Gaidhani, Harwalkar, Bhambere, & Nirgude, 2015). In addition, lyophilized materials have high rehydration properties; therefore, when added to a batter mix, these materials can act as agents that bind the batter ingredients, similar to when grain flour is mixed with water. Fried chicken is a representative food prepared using the deep-frying method. Chicken fingers and chicken tender products (rich in proteins) are made from chicken breast (pectoralis major muscle) and tenderloin (pectoralis minor muscle), respectively (Lee, Camargo, & de Souza Miranda, 2013). In this study we supplemented the frying batter mixture with lyophilized chives and analyzed the quality characteristics (batter mixture viscosity, crispness of the fried batter, proximate composition, calories, coating pickup, pH, frying yield, color, aroma and taste profile, and sensory evaluation) of the resulting batter, fried chicken breasts and tenderloins.

## 2 Materials and methods

### 2.1 Lyophilizing chives

The chives used in this study were purchased from a local market in Chungnam, Korea. These chives were lyophilized to facilitate their supplementation in the batter mix. The chive leaves were first cut into small pieces (3–5 mm) and frozen at −70 °C for 12 h in a deep freezer (TSE320GPD, Thermo Fisher Scientific, Waltham, MA, USA). The frozen chives were lyophilized for 36 h at −121 °C using a lyophilizer (FD12008, Ilshin Bio Base, Yangju, Korea) and were then pulverized. The pH of the lyophilized chives was 5.49 ± 0.03, and the color values were L*: 57.00 ± 1.12, a*: –3.88 ± 0.05, and b*: 13.35 ± 0.37.

### 2.2 Fried chicken breast and tenderloin preparation

The fried chicken breasts and tenderloins were prepared according to Park and Kim, 2021, Adedeji and Ngadi, 2011, with slight modifications. The ingredients of the batter mix for chicken breasts and tenderloins are shown in Supplementary Table 1. The addition of lyophilized chives to the batter mix was as follows: CB0 was the chicken breast-frying batter mix without lyophilized chives, and CB3, CB5, and CB7 were the chicken breast-frying batter mixes supplemented with 3%, 5%, and 7% lyophilized chives, respectively; TL0 was the chicken tenderloin frying batter mix without lyophilized chives, and TL3, TL5, and TL7 were the chicken tenderloin frying batter mixes supplemented with 3%, 5%, and 7% lyophilized chives, respectively. Chicken breasts (pectoralis major muscle) and tenderloins (pectoralis minor muscle) were obtained from Ross broiler chickens (*Gallus domesticus*). For curing, raw chicken breast and tenderloin meat samples were incubated with the curing mixture (0.4% sugar, 0.3% salt, 0.1% monosodium glutamate, 0.2% black pepper powder, 0.1% garlic powder, 11% water; percentages are relative to the raw meat weight) at 4 °C for 24 h in a refrigerator. To prepare the frying batter, the batter mix was mixed with purified water at a ratio of 3:4. The raw meat was dipped into the prepared frying batter for 30 s, removed, and allowed to hang for 25 s. Then, the pieces were deep-fried in soybean oil at 180 °C for 4 min using a fryer (MSM-100 T, Donghwa plant, Korea) and cooled at room temperature (24 °C) for 30 min.

### 2.3 Evaluation of batter mixture viscosity

The viscosity of the batter mixture (12 mL) was measured according to the method described by Park and Kim (2021), using a rotational viscometer (Merlin VR, Rheosys, Hamilton Township, NJ, USA) equipped with a 30-mm cone and a 25-mm co-axial cylinder at 20 °C and 20 rpm for 60 s. The measured viscosity values were averaged and are expressed in Pa·s.

### 2.4 Evaluation of crispness of the fried batter

The crispness of the fried chicken breast and tenderloin batter was measured according to Gallegos-Marin (2020). The crispness was measured at a test speed of 2 mm/s and a test diameter of 10 mm using a texture analyzer (TA1, Lloyd Instruments, FL, USA) equipped with a ball probe (thread size, 6 mm; diameter, 0.25 in.). The measured value was expressed in N.

### 2.5 Evaluation of proximate composition

The proximate composition of the fried chicken breast and tenderloin samples was determined according to the methods described in the Association of Official Analytical Chemists (AOAC, 1990). The moisture, crude protein, crude fat, and ash contents were measured using oven-drying, Kjeldahl, Soxhlet, and dry ashing methods, respectively.

### 2.6 Evaluation of caloric values

The caloric values of the fried chicken breast and tenderloin samples (0.5 g of each) were measured using a bomb calorimeter (C1, IKA, Germany). The bomb calorimeter settings were as follows: water pump temperature of 18.5 °C; water pump speed of 2,800 rpm; and a meat product reference calorific value of 50 cal/L·g. The measured value is expressed in kcal/g.

### 2.7 Evaluation of coating pickup

The coating pickup of the samples was measured according to Martin Xavier et al. (2017). It was determined by measuring the sample weight before and after coating, according to Eq. (1).

(1) $$Coating\;pickup\;\left(\%\right)=\frac{\mathrm{Sample}\;\mathrm{weight}\;\mathrm{after}\;\mathrm{coating}\;(g)}{\mathrm{Sample}\;\mathrm{weight}\;\mathrm{before}\;\mathrm{coating}\;(g)}\times 100$$

### 2.8 Evaluation of pH

To measure the pH of the fried chicken breast and tenderloin samples, the samples (4 g) were mixed with 16 mL of distilled water and homogenized for 1 min using an HMZ-20DN Ultra-Turrax homogenizer (Poolim Tech, Seoul, Korea) at 10,923 × *g*. The pH was measured using a Model S220 pH meter (Mettler-Toledo, Schwerzenbach, Switzerland).

### 2.9 Evaluation of frying yield

The frying yield of the samples were analyzed according to Park and Kim (2021) with some modifications. Sample yields were calculated from the weight of the sample coated batter mixture before and after frying, using Eq. (2).

(2) $$Frying\;yield\;\left(\%\right)=\frac{\mathrm{Weight}\;\mathrm{after}\;\mathrm{frying}\;(g)}{\mathrm{Weight}\;\mathrm{before}\;\mathrm{frying}\;(g)}\times 100$$

### 2.10 Evaluation of color

The CIE general color attributes (lightness, L*; redness, a*; yellowness, b*) of the chicken breast and tenderloin samples were measured using a CR-10 color reader (Minolta, Tokyo, Japan) with a white standard plate (CIE L*: +97.83; CIE a*: −0.43; CIE b*: +1.98) as a reference.

### 2.11 Evaluation of aroma profiles

The aroma profiles of the fried chicken breast and tenderloin samples were analyzed using a Heracles II electronic nose (Alpha MOS, Toulouse, France). The electronic nose analysis conditions were as follows: 1 g of the sample into a 20-mL vial, flow rate of 250 mL/min, acquisition time of 120 s, headspace temperature of 60 °C, and quantity injection of 2.5 mL. Before principal component analysis (PCA), the sensitivity of each electronic nose sensor was measured to determine the rate of change between the resistance values of the volatile compounds and those of the air. The values of the sensitivity measured using the electronic nose sensor and each quantification peak of volatile components are presented in a chromatogram using the Alpha software program (for an electronic nose; Alpha MOS, Toulouse, France). The aromatic profiles of volatile compounds in the measured samples were obtained by PCA using the Alpha software program, and the differences in the aromatic profiles between samples are presented as plot coordinates. The classified aroma pattern was reported as the primary component value (PC1) and secondary component value (PC2).

### 2.12 Evaluation of taste profile

The taste profiles of the fried chicken breast and tenderloin samples were measured using an Astree electronic tongue (Alpha MOS, Toulouse, France). The sourness, saltiness, and umami of the samples were measured using 0.1 M HCl, 0.1 M NaCl, and 0.1 M MSG as reference materials for the electronic tongue sensor, respectively. The samples (4 g) were mixed with 16 mL of distilled water and homogenized for 1 min using an Ultra-Turrax homogenizer. Then, the homogenate was filtered. The taste profile of the filtrate (diluted with distilled water at a ratio of 1:100) was analyzed using the electronic tongue (acquisition duration, 120 s; acquisition period, 1 s). The taste sensitivity of the electronic tongue was analyzed using the Alpha soft program for electronic tongues, and expressed as AHS (sourness), PKS, CTS (saltiness), NMS (umami), CPS, ANS, and SCS. The taste profiles, of the measured sample sensitivity of each sensor, were obtained by performing a PCA using the Alpha software program for electronic tongues, and the differences in taste profiles between samples are presented as plot coordinates. The classified taste pattern is reported as the primary component value (PC1) and the secondary component value (PC2).

### 2.13 Sensory evaluation

Sensory evaluation was performed according to Park and Kim, 2021, Gouyo et al., 2020. Twenty-four sensory panelists used a basic taste identification test to evaluate the sensory characteristics of the fried samples. Panelists consisted of undergraduate and graduate students, majoring in food science related fields, who were trained using commercially available fried chicken fingers and chicken tender products for seven days (1 h session per day); to ensure familiarization with the sensory characteristics, of fried chicken breast and tenderloin, to be evaluated. The color, flavor, crispness, juiciness, off-flavor, and overall acceptability of the samples were evaluated using a 10-point descriptive scale (color appearance: 1 = extremely undesirable, 10 = extremely desirable; flavor: 1 = extremely inadequate, 10 = extremely adequate; sensory crispness: 1 = extremely soft, 10 = extremely crispy; juiciness: 1 = extremely dry, 10 = extremely juicy; off-flavor: 1 = extremely off-flavor, 10 = no off-flavor; and overall acceptability: 1 = extremely unacceptable, 10 = extremely acceptable). The sensory evaluation traits analyzed in this study were selected from the sensory evaluation traits reported in Park and Kim, 2021, Gouyo et al., 2020. The sensory evaluation procedure was approved by the Ethics Committee of Kongju National University, Korea (Authority No: KNU_IRB_2020-40).

### 2.14 Statistical analysis

At least three independent trials were performed for all analyses. All data (except the aromatic and taste profile analyses) were statistically analyzed using analysis of variance (ANOVA) for all variables, followed by Duncan’s multiple range test (*P* < 0.05) and the general linear model in SAS version 9.3 (SAS Institute, Cary, NC, USA). All the results (except for viscosity and crispness) the breast and tenderloin fried chicken groups were statistically analyzed.

## 3 Results and discussion

### 3.1 Effect of lyophilized chive supplementation on the viscosity of frying batter and crispness of the fried batter

**Fig. 1:**
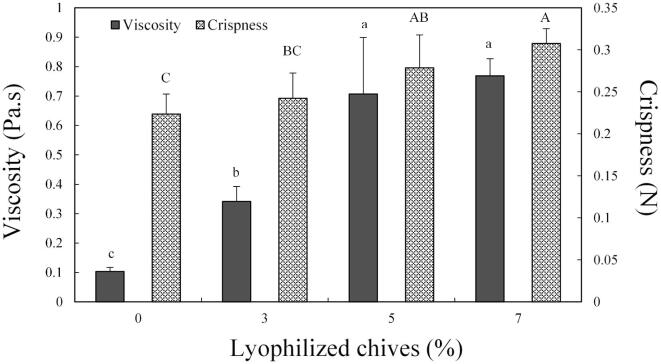
Viscosity of non-fried batter and crispness of fried batter with various levels of lyophilized chives. ^a-c, A-C^ Means on the same bar with different letters are significantly different (*P* < 0.05). Experimental results were derived from five repeated analyses.

Fig. 1 shows the viscosity of the batter mixture supplemented with the different amounts of lyophilized chives. The viscosity of the chicken frying batter (0.10–0.77 Pa·s) varied depending on the amount of lyophilized chives in the batter. The lyophilized chive-supplemented batter exhibited significantly higher viscosity than the CB0 AND TL0 batters (*P* < 0.05). Additionally, the viscosity of the 5% and 7% lyophilized chive-supplemented batter was significantly higher than that of the 3% lyophilized chive-supplemented batter and CB0 and TL0 batters (P < 0.05). There was no significant difference between the 5% and 7% chive groups. With hydrated materials, there is a limit to aqueous solvation under a constant volume of water (Biswas, Tse, Tokmakoff, & Voth, 2015). Accordingly, the samples were judged to be saturated when the amount of lyophilized chives added to the batter mixture prepared exceeded 5%. Supplementation with chives increased the viscosity of the batter by up to seven times. This high viscosity enables a stable batter coating as it decreases the rate of batter falling off from the coated raw meat (Varela & Fiszman, 2011). Additionally, a stable batter coating prevents the formation of gaps in the fried coat surrounding the raw meat and increases the yield of fried food (Park & Kim, 2021). Furthermore, a high viscosity indicates a homogenous mixing of additives. In the food industry, binding agents, such as whey protein, are added to increase the viscosity of the mixture (Castro et al., 2017). In this study, viscosity was significantly increased by adding lyophilized chives to the batter, therefore, the lyophilized chives can function as a binding agent in the batter. The increased viscosity of the batter was attributed to the rehydration properties of the lyophilized materials (Kurek, Ščetar, & Galić, 2017). Therefore, the high viscosity is result of the increased absorption of water by the lyophilized chives in the batter mix.

Crispness values of the fried batter are shown in Fig. 1. The crispness of the batter increased along with the amount of lyophilized chives in the batter (0.22–0.31 N). The caramelization of reducing sugars in the lyophilized chives contributes to crispness. In general, when reducing sugars are caramelized, their physical properties significantly change. When heated to high temperatures, the sugars melt to a liquid state. After heating, the sugars harden again. It is known that the hardness of reducing sugar mixes (that includes some other materials) is further increased after the caramelization process is completed (Badía, Kastelijn, Scheerder, & Leiza, 2020). Onions are representative caramelization materials. The chives used in this study are characterized by a high content of reducing sugars, as they are hybrids of spring onions and onions (Lee et al., 2009). Therefore, heating to a high temperature of 180 °C increases the caramelization of the reducing sugars in chives and consequently increases the hardness of the batter. Additionally, non-Newtonian fluids, such as the batter, tend to exhibit a higher binding strength between ingredients in the batter mixture, which further increases the viscosity (Nazaruddin & Azis, 2019). The firmness of the fried batter increased with the increase in the binding strength between the batter components.

### 3.2 Effect of lyophilized chive supplementation on coating pickup rates, pH, and frying yield

The coating pickup rates for chicken breast and tenderloin batter mix supplemented with lyophilized chives are shown in Table 1. The coating pickup rates of the chicken breast batter mix increased with the amount of lyophilized chives in the batter (*P* < 0.05). The coating pickup rates were significantly higher in the TL5 and TL7 compared to those of the TL0 and TL3 groups (*P* < 0.05). These results are related to the increase in the viscosity of the batter (Varela & Fiszman, 2011). In this study, it was confirmed that as the amount of lyophilized chives in the batter increased, the viscosity of the batter mixture increased, the rate of batter falling off the coated raw meat decreased, and consequently the coating pickup rate increased (Martínez, Sanz, & Gómez, 2015). As mentioned above, there is a close relationship between viscosity and coating pickup, but in the case of tenderloin, there was no significant difference between the TL5 and TL7 groups with respect to viscosity, whereas the CB5 breast sample group had a lower coating pickup value than the CB7 group. This is probably because non-hydrated lyophilized chives remain on the breast surface because they have a larger surface area than tenderloin, which increases the batter pickup rate.

**Table 1:** Batter coating pickup, pH, and frying yield of batter with chicken breast and tenderloin formulated with various levels of lyophilized chives.

Treatments		Coating pickup (%)	pH	Frying yield (%)
Breast1)	CB0	105.35 ± 1.38^d^	6.25 ± 0.01^a^	70.83 ± 3.73^b^
	CB3	109.88 ± 2.57^c^	6.23 ± 0.04^ab^	71.77 ± 4.66^b^
	CB5	114.29 ± 1.94^b^	6.21 ± 0.01^bc^	89.61 ± 7.21^ab^
	CB7	121.18 ± 1.02^a^	6.19 ± 0.01^c^	93.52 ± 3.17^a^

Tenderloin2)	TL0	106.15 ± 1.25^B^	6.31 ± 0.02^A^	63.75 ± 2.55^C^
	TL3	108.11 ± 4.47^B^	6.31 ± 0.01^AB^	66.06 ± 6.42^BC^
	TL5	118.90 ± 4.21^A^	6.30 ± 0.01^AB^	72.77 ± 1.19^AB^
	TL7	121.89 ± 3.94^A^	6.28 ± 0.01^B^	78.08 ± 2.29^A^

Data are shown as means ± SD.

^a-d^ Means on the same column of chicken breast samples with different letters are significantly different (*P* < 0.05).

^A-C^ Means on the same column of chicken tenderloin samples with different letters are significantly different (*P* < 0.05).

Experimental results were derived from three repeated analyses.

1) CB0: chicken breast batter without lyophilized chives; CB3: chicken breast batter with 3% lyophilized chives; CB5: chicken breast batter with 5% lyophilized chives; CB7: chicken breast batter with 7% lyophilized chives.

2) TL0: chicken tenderloin batter without lyophilized chives; TL3: chicken tenderloin batter with 3% lyophilized chives; TL5: chicken tenderloin batter with 5% lyophilized chives; TL7: chicken tenderloin batter with 7% lyophilized chives.

Table 1 shows the pH values of fried chicken breast and tenderloin samples. The pH of both the breast and tenderloin samples decreased with the amount of lyophilized chives in the batter, which can be attributed to the low pH of the lyophilized chives (5.49 ± 0.03). The pH of plants or plant extracts supplemented to the meat products determines the pH of the final product (Shah, Bosco, & Mir, 2014). Therefore, batter supplementation with lyophilized chives affected the pH of the meat products coated with batter. The pH value is an important quality standard for deep-fried products. Deep-frying using ingredients with low pH decreases the pH of the oil, which can increase the rancidity of the oil and fried products (Stojanovska & Tomovska, 2015). In this study, the pH decreased as the amount of lyophilized chives added increased. Therefore, it is necessary to limit the addition of chives to an appropriate level.

In the process whereby raw meat is dipped into the batter mixture, the viscosity of the batter mixture may decrease because of the moisture on the surface of the raw meat. Therefore, the batter mixture could be divided out from meat during the frying process, accordingly, analyzed the frying yield (Table 1). The frying yield in both the chicken breast and tenderloin samples increased with the amount of lyophilized chives in the batter. In fried products, a stable batter coating prevents the separation of the batter from the meat during frying and ensures a uniform outer coat, which prevents exudation and excessive drying of the meat juice generated during the heating process (Park & Kim, 2021). In this study, it was confirmed that the viscosity of frying batter and coating pickup rate increased as the amount of lyophilized chives added increased. Accordingly, the supplementation of lyophilized chives to the batter mixture enabled a stable batter coating and consequently increased the frying yield. The pH of meat products is associated with the water holding capacity and water loss during heating. As the pH approaches 5.0–5.2, which is the isoelectric point of proteins, the water holding capacity decreases and the moisture exudation during heating increases, resulting in increased water loss (Chen et al., 2019). In this study, the frying yield increased even though the pH decreased with the addition of lyophilized chives to the batter. This can be attributed to the stable coating of the batter supplemented with lyophilized chives on the raw meat, which decreased the exudation of oil and moisture.

### 3.3 Effect of lyophilized chive supplementation on proximate composition and calorie content

Table 2 shows the proximate compositions of the fried chicken breasts and tenderloin samples according to the amount of lyophilized chives in the frying batter. The moisture content did not significantly vary among the CB0, CB3, CB5, and CB7 groups. The protein content in the CB7 group was significantly lower than those in the other groups (*P* < 0.05). The fat contents in the CB5 and CB7 groups were significantly higher than those in the CB0 and CB3 groups (*P* < 0.05). The ash content increased with the amount of lyophilized chives in the batter. The protein content in the tenderloin samples decreased with the amount of lyophilized chives in the batter. The moisture, fat, and ash contents in the TL5 and TL7 groups were significantly higher than those in the TL0 and TL3 groups (*P* < 0.05). These changes in the proximate composition are associated with the increased coating rates of the batter (Zhang, Li, Ding, & Fan, 2016). The coating pickup rate increased with the amount of lyophilized chives in the batter. The amount of edible oil absorbed in the batter increases with the amount of batter coating the meat surface. Consistently, the fat and ash contents increased with the amount of lyophilized chives in the batter. In contrast, the protein content of the tenderloin samples decreased with the amount of lyophilized chives in the batter. Plant extracts, such as lyophilized chives, mostly consist of dietary fiber (Veiga, Costa, Sliva, & Pintado, 2020), accordingly, it is thought that the ash content increases as the amount of lyophilized chives in the batter increases. The decrease in protein content is considered to be related to the fat content among the proximate composition ratios of the samples. The reason for the decrease in protein content is that the fat content proportion increased in the proximate composition in relative terms, which meant that the protein content proportion decreased. However, the effect of lyophilized chives and batter on the protein content of chicken meat was not considered.

**Table 2:** Proximate composition, calorie, and color of fried batter with chicken breast and tenderloin formulated with various levels of lyophilized chives.

Treatments		Proximate composition				Calorie (kcal/g)	Color		
		Water (%)	Protein (%)	Fat (%)	Ash (%)		Lightness (L*)	Redness (a*)	Yellowness (b*)
Breast1)	CB0	38.50 ± 0.26	23.52 ± 0.48^a^	32.04 ± 0.19^b^	1.44 ± 0.03^c^	1.84 ± 0.01^b^	53.00 ± 1.67^a^	14.22 ± 1.27^a^	33.47 ± 0.55^a^
	CB3	38.68 ± 1.32	24.00 ± 0.27^a^	32.51 ± 0.69^b^	1.48 ± 0.01^bc^	1.82 ± 0.05^b^	52.24 ± 4.90^b^	12.00 ± 1.45^b^	30.43 ± 0.55^b^
	CB5	38.09 ± 0.01	23.63 ± 0.38^a^	33.55 ± 0.18^a^	1.49 ± 0.02^b^	2.03 ± 0.04^a^	43.57 ± 1.76^b^	4.88 ± 0.78^b^	26.38 ± 1.17^b^
	CB7	39.42 ± 0.21	22.12 ± 0.31^b^	33.74 ± 0.52^a^	1.57 ± 0.04^a^	1.97 ± 0.04^a^	34.36 ± 1.86^b^	3.12 ± 0.65^c^	18.78 ± 2.42^b^

Tenderloin2)	TL0	43.36 ± 1.17^B^	22.47 ± 0.54^A^	33.35 ± 0.38^B^	1.57 ± 0.02^B^	2.49 ± 0.03^C^	51.30 ± 2.35^A^	17.02 ± 0.65^A^	32.92 ± 3.35^A^
	TL3	44.03 ± 1.18^B^	22.25 ± 0.25^A^	32.68 ± 0.49^B^	1.57 ± 0.02^B^	2.62 ± 0.10^B^	42.80 ± 0.20^B^	6.76 ± 1.13^B^	25.00 ± 2.36^B^
	TL5	47.82 ± 0.52^A^	21.15 ± 0.41^AB^	35.91 ± 0.25^A^	1.67 ± 0.01^A^	2.87 ± 0.01^A^	43.18 ± 3.65^B^	6.84 ± 1.70^B^	25.36 ± 3.74^B^
	TL7	47.65 ± 0.72^A^	21.79 ± 0.27^B^	35.18 ± 0.40^A^	1.65 ± 0.01^A^	2.84 ± 0.07^A^	41.38 ± 1.22^B^	1.52 ± 0.69^C^	23.02 ± 1.51^B^

Data are shown as means ± SD.

^a-b^ Means on the same column of chicken breast samples with different letters are significantly different (*P* < 0.05).

^A-C^ Means on the same column of chicken breast samples with different letters are significantly different (*P* < 0.05).

The proximate composition and colorie experimental results were derived from three repeated analyses.

The color experimental results were derived from three repeated analyses.

1) CB0: chicken breast batter without lyophilized chives; CB3: chicken breast batter with 3% lyophilized chives; CB5: chicken breast batter with 5% lyophilized chives; CB7: chicken breast batter with 7% lyophilized chives.

2) TL0: chicken tenderloin batter without lyophilized chives; TL3: chicken tenderloin batter with 3% lyophilized chives; TL5: chicken tenderloin batter with 5% lyophilized chives; TL7: chicken tenderloin batter with 7% lyophilized chives.

The caloric values of the fried chicken breast and tenderloin samples are listed in Table 2. The CB5 and CB7 groups exhibited significantly higher caloric values than the CB0 and CB3 groups (*P* < 0.05). Therefore, the caloric values in the tenderloin samples increased with the amount of lyophilized chives in the batter. This increase in the caloric values can be attributed to the increase in the amount of batter absorbing edible oil during frying, as evidenced by the increased coating pickup rate in the lyophilized chive-supplemented groups. Thus, batter fall-off rates are inversely proportional to the caloric values of battered and breaded foods. Additionally, the constituents of the fried products are related to the oil absorption rate of the batter, and are affected by various factors, such as frying time, temperature, product shape, and product surface shape after frying (Dehghannya & Ngadi, 2021). Therefore, stably formed batter is believed to exhibit enhanced oil absorption and increased caloric values. However, the incidence of diseases, such as obesity and hypertension, caused in part by the consumption of fried foods with high caloric values, is increasing in the modern population (Zhang et al., 2016). Thus, the caloric values of fried products must be adjusted to a level that does contribute to such pathological conditions.

### 3.4 Effect of chive supplementation on color

The visual evaluation of the fried products depends on the batter that coats the products (Rozzamri et al., 2020). In this study, the chives were subjected to lyophilization, which did not adversely affect the properties of the raw materials (Różyło, 2020). The natural color of the chives was maintained even after lyophilization (L*, 57.00; a*, −3.88; b*, 13.35). Chives leaves are composed of a complex mixture of phytochemicals, including chlorophyll and carotenoid pigments (Veiga et al., 2020). Chives are greenish in color, and colors like blue and green can give the perception of “healthy products” when evaluating the appearance of foods (Schuldt, 2013). The color attributes of the fried chicken breast and tenderloin samples coated with batter supplemented with lyophilized chives are shown in Table 2. Compared with those in the CB0 group, the lightness, redness, and yellowness were significantly lower in the CB3, CB5, and CB7 groups (*P* < 0.05). Similarly, the lightness, redness, and yellowness of the TL3, TL5, and TL7 groups were significantly lower than those in the TL0 group (*P* < 0.05). In this study, the greenish chromaticity of the lyophilized chives markedly affected the color attributes of the chicken breast and tenderloin batter mixtures. During the deep-frying process, the high starch content of the batter promotes a Maillard reaction. Additionally, starch absorbs oil and imparts a light brown color to the batter (Dehghannya & Ngadi, 2021). Lyophilized chives exhibited a slightly green color even after deep-frying. Thus, the fried batter exhibited differential color intensities depending on the amount of lyophilized chives in the batter. Food products are usually positively evaluated when consumers can confirm the use of functional additives based on the appearance (Fiorentini, Kinchla, & Nolden, 2020). However, fried products are generally golden-brown in color (Rozzamri et al., 2020). Hence, cool colors are not suitable for evaluating the appearance of meat products. As the supplementation of lyophilized chives changed the appearance of the fried chicken breast and tenderloin, the amount of lyophilized chives must be determined to optimize the appearance.

### 3.5 Effect of lyophilized chive supplementation on the aromatic and taste profiles

**Fig. 2:**
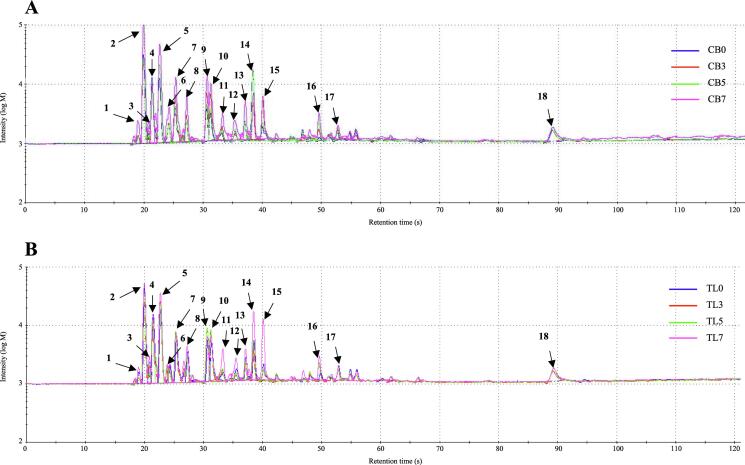

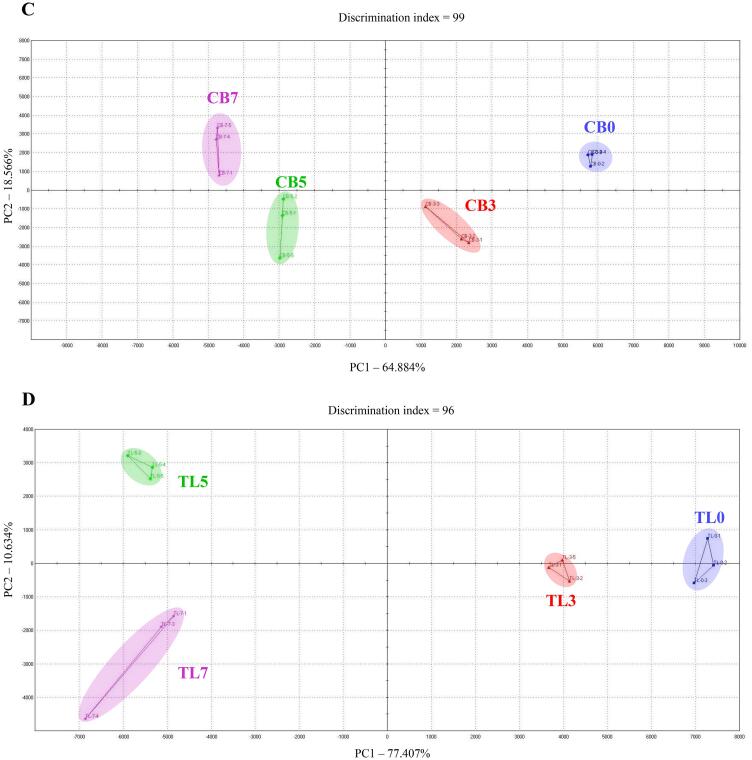
Volitile compounds of fried chicken breast (A) tenderloin (B) samples, and principal component analysis (PCA) plot for aroma profile of fried chicken breast (C) tenderloin (D) samples. Experimental results were derived from five repeated analyses. Fig. 2-A and B peaks are reported in order of elution: 1: Methanethiol; 2: Propan-2-one; 3: 2-methylpropanal; 4: 2-mercaptoethanol; 5: 2-methylfuran; 6: 1-propanol, 2-methyl-; 7: Butan-2-one; 8: Thiophene; 9: 1-penten-3-one; 10: 2,3-pentanedione; 11: Dimethyl disulfide; 12: 1-Chloropentane; 13: Methyl 2-methylbutanoate; 14: 2-hexanol; 15: 2-acetyl-1-pyrroline; 16: Z-3-Hexen-1-ol; 17: 2,4,5-trimethylthiazole; 18: Methyl eugenol, CB0: chicken breast batter without lyophilized chives; CB3: chicken breast batter with 3% lyophilized chives; CB5: chicken breast batter with 5% lyophilized chives; CB7: chicken breast batter with 7% lyophilized chives. TL0: chicken tenderloin batter without lyophilized chives; TL3: chicken tenderloin batter with 3% lyophilized chives; TL5: chicken tenderloin batter with 5% lyophilized chives; TL7: chicken tenderloin batter with 7% lyophilized chives.

Fig. 2-A and B shows the volatile compounds in the fried chicken breast and tenderloin samples. For both the chicken breast and tenderloin samples, the flavor compound peaks for 2-methylfuran, thiophene, dimethyl disulfide, 1-chloropentane, methyl 2-methylbutanoate, 2-acetyl-1-pyrroline, and z-3-hexen-1-ol increased with the lyophilized chive content in the batter (peaks numbered 5, 8, 11, 12, 13, 15, and 16, respectively). In particular, the flavors corresponding to thiophene, dimethyl disulfide, 1-chloropentane, methyl 2-methylbutanoate, and z-3-hexen-1-ol are responsible for the aroma characteristics produced by garlic, cabbage, onion, and green plants; and the earthy, fruity, and sweet flavors. These aromas and flavors are thought to be directly affected by lyophilized chives. The 2-methylfuran and 2-acetyl-1-pyrroline compounds produce aromatic sensations like such as burnt, sweet gassy, roast, and nutty, and overheated meat-like flavor. It is thought that the reducing sugars contained in lyophilized chives are caramelized, thus generating these flavors. Fig. 2-C and D shows the aromatic profiles in a PCA plot based on the volatile compounds in the fried chicken breast and tenderloin samples. In the breast samples, PC1 (x-axis) and PC2 (y-axis) were 64.884% and 18.566%, respectively. In the tenderloin samples, PC1 and PC2 were 77% and 10%, respectively. Therefore, the difference in flavor between the breast and tenderloin samples is determined according to the distance on the x-axis corresponding to PC1 (Park, Seol, & Kim, 2020). The results of PCA revealed that the CB0 and CB3 groups exhibited clearly differentiated flavors, and that the flavor of CB5 was different from those of CB0 and CB3. However, the difference in flavor characteristics between the CB7 and CB5 groups was not greater than those between the CB0, CB3, and CB5 groups. Similar results were obtained for the flavor characteristics of tenderloin samples from the TL5 and TL7 groups, which exhibited similar flavor. Chives contain high levels of reducing sugars, which leads to the Maillard reaction, which is a non-enzymatic browning reaction, occurring after heating (Dehghannya & Ngadi, 2021). Flavor compounds that impart this unique flavor, such as pyrroles, thiophenes, furans, and pyrazines, are formed through the generation of various volatile compounds in this process (Yuliarti, Kovis, & Yi, 2021). Therefore, the formation of flavor compounds through the Maillard reaction is affected by the amount of lyophilized chives in the batter, which determines the flavor of the samples.

**Fig. 3:**
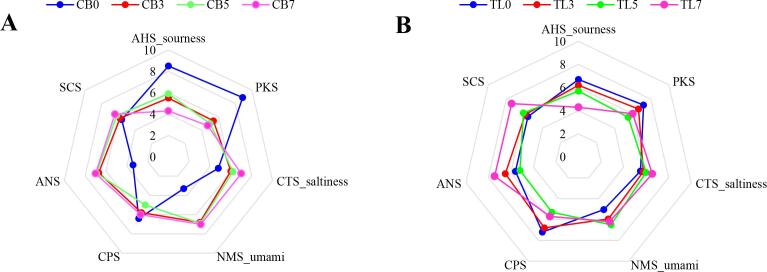

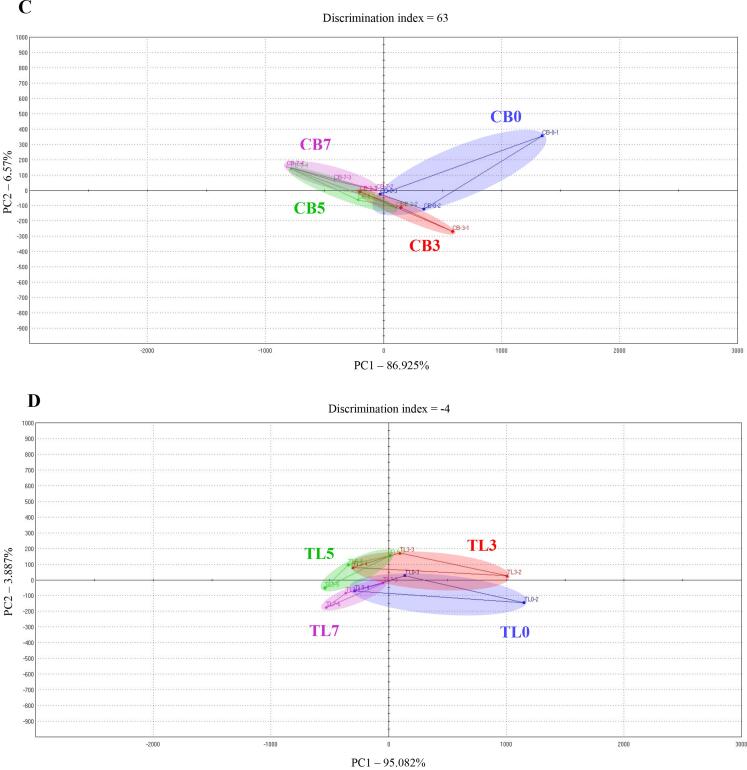
Radial graph for taste attributes of fried chicken breast (A) tenderloin (B) samples, and principal component analysis (PCA) plot for taste profile of fried chicken breast (C) and tenderloin (D) samples. Experimental results were derived from five repeated analyses. CB0: chicken breast batter without lyophilized chives; CB3: chicken breast batter with 3% lyophilized chives; CB5: chicken breast batter with 5% lyophilized chives; CB7: chicken breast batter with 7% lyophilized chives. TL0: chicken tenderloin batter without lyophilized chives; TL3: chicken tenderloin batter with 3% lyophilized chives; TL5: chicken tenderloin batter with 5% lyophilized chives; TL7: chicken tenderloin batter with 7% lyophilized chives.

The electronic tongue taste sensor reactivity values of the samples are shown in Fig. 3-A and B. Sourness (AHS) decreased (breast: 8.5–4.3; tenderloin: 6.7–4.3), whereas saltiness (CTS) increased with the amount of lyophilized chives in the batter (breast: 4.8–7; tenderloin: 5.5–6.5). However, in the case of sourness, the CB7 and TL7 groups seemed to have clearly lower values than the other groups, whereas saltiness was similar between the lyophilized chive-supplemented groups. Lyophilized chive-supplemented groups exhibited higher umami profiles than the CB0 and TL0 groups (breast: 3.3–7; tenderloin: 5.1–6.2). However, the CB3 and CB5, and TL0 and TL5 groups had similar umami values. The taste profiles were obtained using a PCA based on the reactivity of all the sensors, which was derived from the electronic tongue analysis results of the individual samples. The analysis results are shown in Fig. 3-C and B. Similar to the aromatic profile PCA plot, PC1 (breast: 86.925%; tenderloin: 95.082%) showed greater values than PC2 (breast: 6.57%; tenderloin: 3.887%). Therefore, among the chicken breast groups shown on the x-axis, CB0 and CB3 had quite similar taste profiles, while CB5 and CB7 showed slightly different taste profiles from CB0 and CB3. The tenderloin groups showed no clear difference in taste profiles among the samples, but showed similar trends to those of the chicken breast groups. Glutamic acid, which is a product of the Maillard reaction in chives during frying, is a free amino acid that contributes to the umami taste (Zou, Gao, He, & Yang, 2018). Thus, lyophilized chives may enhance the umami taste. The decreased sourness in lyophilized chive-supplemented groups appears to be due to the pyruvate in chives, which is characteristic to the genus *Allium*, and pyruvic acid exhibits irritant and pungent properties (Ninomiya, 2015). The sourness decreased owing to these properties of pyruvic acid. Supplementation with lyophilized chives affected the taste and flavor profiles of the fried chicken breast and tenderloin samples. However, the 5% and 7% lyophilized chive-supplemented groups had similar aromatic and taste profiles. This indicated that the supplementation of lyophilized chives at amounts greater than 5% did not markedly improve the flavor and taste profiles.

### 3.6 Sensory evaluation

The sensory evaluation results for the different fried chicken breast and tenderloin groups are shown in Table 3. The CB3 and CB5 groups received the highest scores for appearance, color, sensory crispness, and overall acceptability (*P* < 0.05). The flavor of the CB5 group was significantly higher than that of the other groups (*P* < 0.05). The juiciness of the lyophilized chive-supplemented groups was significantly higher than that of the CB0 group (*P* < 0.05), whereas the off-flavor of the CB7 group was significantly lower than that of the other groups (*P* < 0.05). Among tenderloin samples, the TL3 and TL5 groups had the highest scores for appearance, color, flavor, sensory crispness, and overall acceptability (*P* < 0.05). The juiciness and off-flavor of the tenderloin groups were similar to the evaluation results of the chicken breast groups. The juiciness of the lyophilized chive-supplemented groups was significantly higher than that of the TL0 group (*P* < 0.05), and the TL7 group had the lowest off-flavor score (*P* < 0.05). In this study, the difference in the chromaticity of the samples can be attributed to the differences in the amount of greenish lyophilized chives (L*, 57.00; a*, −3.88; b*, 13.35) in the batter. The CB7 and TL7 groups received the lowest scores for redness, which may have been determined as unfamiliar by the evaluation panel. Fried chicken is generally known to be golden-brown in color. As the natural color of the chives used in this study is green, enhanced supplementation of lyophilized chives in the batter may lead to rejection. The flavor and off-flavor evaluation scores were different from those evaluated using the electronic nose analysis. The CB7 and TL7 groups showed low flavor and off-flavor scores. The results of the electronic nose analysis revealed that the CB5 and CB7 groups as well as the TL5 and TL7 groups exhibited similar flavors. The flavor of the chives used as a condiment vegetable is pyruvic acid, which has a strong odor (Barboza et al., 2020). Hence, the sensory properties of chives significantly change even though there is no significant difference in the composition of pyruvic acid among the main flavor components. The CB3, CB5, TL3, and TL5 groups received excellent evaluation scores. This can be attributed to the decreased fall-off rate of the batter supplemented with lyophilized chives and the formation of a uniform and solid layer after frying. However, in a previous study, Voong, Norton, Mills, and Norton (2018) reported that high concentrations of batter coat require a higher level of breaking stress to chew the fried product. Thus, batter with an appropriate concentration should be prepared. This can explain the low sensory evaluation scores of CB7 and TL7, even though they exhibited the highest crispness. Supplementation with lyophilized chives enabled the formation of a uniform and solid batter, which meant that juiciness of the CB3, CB5, CB7, TL3, TL5, and TL7 groups was higher than that of the CB0 and TL0 groups. This is because batter mixes with these characteristics would form a uniform layer during frying, thereby preventing the exudation of moisture from the raw material (Mba, Dumont, & Ngadi, 2015). The results of sensory evaluation of the breast and tenderloin samples indicated that supplementation with 3% and 5% lyophilized chives was optimal. However, the taste of the 3% lyophilized chive-supplemented group was relatively low. Thus, supplementation with 5% lyophilized chives can be considered optimal for batter preparation.

**Table 3:** Sensory evaluation of fried chicken breast and tenderloin formulated with various levels of lyophilized chives.

Treatments		Color	Flavor	Sensory crispness	Juiciness	Off-flavor	Overall acceptability
Breast1)	CB0	7.63 ± 0.49^c^	7.88 ± 0.80^c^	8.13 ± 0.61^b^	8.00 ± 1.14^b^	9.24 ± 0.66^a^	7.94 ± 0.83^c^
	CB3	9.00 ± 0.51^a^	8.63 ± 0.71^b^	9.00 ± 0.55^a^	8.75 ± 0.99^a^	9.25 ± 0.68^a^	9.13 ± 0.80^a^
	CB5	9.25 ± 0.68^a^	9.25 ± 0.68^a^	9.00 ± 0.84^a^	8.86 ± 0.85^a^	9.38 ± 0.88^a^	9.50 ± 0.51^a^
	CB7	8.25 ± 0.68^b^	8.50 ± 1.02^b^	8.13 ± 1.30^b^	8.71 ± 0.90^a^	8.88 ± 1.08^b^	8.64 ± 0.98^b^

Tenderloin2)	TL0	7.75 ± 0.68^C^	7.38 ± 0.88^C^	7.75 ± 0.85^B^	7.88 ± 0.80^B^	9.13 ± 0.81^A^	7.94 ± 0.83^B^
	TL3	8.88 ± 0.80^A^	8.75 ± 0.68^A^	9.00 ± 1.02^A^	8.88 ± 0.80^A^	9.25 ± 0.68^A^	9.25 ± 0.68^A^
	TL5	8.88 ± 0.80^A^	9.13 ± 0.80^A^	9.25 ± 0.68^A^	8.88 ± 0.80^A^	9.13 ± 0.95^A^	9.25 ± 0.68^A^
	TL7	8.38 ± 0.88^B^	8.25 ± 0.99^B^	8.00 ± 1.02^B^	8.75 ± 0.85^A^	8.50 ± 1.02^B^	8.25 ± 0.85^B^

Data are shown as means ± SD.

^a-b^ Means on the same column of chicken breast samples with different letters are significantly different (*P* < 0.05).

^A-B^ Means on the same column of chicken tenderloin samples with different letters are significantly different (*P* < 0.05).

Experimental results were derived from sensory evaluations by 24 sensory panelists.

1) CB0: chicken breast batter without lyophilized chives; CB3: chicken breast batter with 3% lyophilized chives; CB5: chicken breast batter with 5% lyophilized chives; CB7: chicken breast batter with 7% lyophilized chives.

2) TL0: chicken tenderloin batter without lyophilized chives; TL3: chicken tenderloin batter with 3% lyophilized chives; TL5: chicken tenderloin batter with 5% lyophilized chives; TL7: chicken tenderloin batter with 7% lyophilized chives.

## 3 Conclusion

In this study, fried chicken breasts and tenderloins were prepared using a batter supplemented with lyophilized chives. Supplementation with lyophilized chives increased the viscosity of the batter and crispness of the fried products. The 5% and 7% lyophilized chive-supplemented groups exhibited similar viscosities and crispness. Additionally, the frying yield, aromatic profile, and taste profile of the fried chicken breasts and tenderloins were similar between the 5% and 7% lyophilized chive-supplemented groups. Regarding sensory evaluation, the CB5 and TL5 groups received higher scores, from the panelists, than the other groups. These results suggest that 5% lyophilized chive supplementation is optimum for the preparation of batter for frying chicken breasts and tenderloins.

## Ethics Approval

The sensory evaluation was approved by the Ethics Committee of Kongju National University, South Korea (Authority No: KNU_IRB_2020-40).

## Funding

This research was supported by Basic Science Research Program through the National Research Foundation of Korea (KRF) funded by the Ministry of Education (2018R1D1A1B07049938).

### CRediT authorship contribution statement

**Sin-Young Park:** Formal analysis, Investigation, Methodology, Software, Visualization, Writing – original draft. **Hack-Youn Kim:** Conceptualization, Data curation, Funding acquisition, Project administration, Resources, Supervision, Validation, Writing – review & editing.

## Declaration of Competing Interest

The authors declare that they have no known competing financial interests or personal relationships that could have appeared to influence the work reported in this paper.
