# Supplementary material for: Effect of lyophilized chive (Allium wakegi Araki) supplementation to the frying batter mixture on quality attributes of fried chicken breast and tenderloin
Source: Food Chem X. 2022 Jan 17;13:100216. doi: 10.1016/j.fochx.2022.100216 (PMC9039885; doi:10.1016/j.fochx.2022.100216)
Supplement: Supplementary data 1 [file mmc1.docx]

Supplementary Table 1. Ingredient of batter mix added with various amounts of lyophilized chives

| Ingredients (g) | Lyophilized chives | | | |
| --- | --- | --- | --- | --- |
|  | 0% | 3% | 5% | 7% |
| Wheat powder | 188 | 188 | 188 | 188 |
| Corn flour | 133.2 | 133.2 | 133.2 | 133.2 |
| Rice powder | 20.8 | 20.8 | 20.8 | 20.8 |
| Potato flour | 20.8 | 20.8 | 20.8 | 20.8 |
| Salt | 2 | 2 | 2 | 2 |
| Sugar | 4 | 4 | 4 | 4 |
| Baking powder | 4 | 4 | 4 | 4 |
| Black pepper powder | 2 | 2 | 2 | 2 |
| Monosodium glutamate | 17.2 | 17.2 | 17.2 | 17.2 |
| Onion powder | 4 | 4 | 4 | 4 |
| Garlic powder | 4 | 4 | 4 | 4 |
| Lyophilized chives | - | 12 | 20 | 28 |

Samples batter mix were mixed with water to a ratio of 3 : 4.
